# Polyphosphate as a Target for Interference With Inflammation and Thrombosis

**DOI:** 10.3389/fmed.2019.00076

**Published:** 2019-04-12

**Authors:** Reiner K. W. Mailer, Lorena Hänel, Mikel Allende, Thomas Renné

**Affiliations:** Institute of Clinical Chemistry and Laboratory Medicine, University Medical Center Hamburg-Eppendorf, Hamburg, Germany

**Keywords:** polyphosphate, factor XII, hereditary angioedema, thrombosis, inflammation, coagulation, immune activation, vascular permeability

## Abstract

Activated platelets and mast cells expose the inorganic polymer, polyphosphate (polyP) on their surfaces. PolyP initiates procoagulant and proinflammatory reactions and the polymer has been recognized as a therapeutic target for interference with blood coagulation and vascular hyperpermeability. PolyP content and chain length depend on the specific cell type and energy status, which may affect cellular functions. PolyP metabolism has mainly been studied in bacteria and yeast, but its roles in eukaryotic cells and mammalian systems have remained enigmatic. In this review, we will present an overview of polyP functions, focusing on intra- and extracellular roles of the polymer and discuss open questions that emerge from the current knowledge on polyP regulation.

## Polyphosphate Metabolism

Inorganic polyphosphate (polyP) is abundantly found in every cell in nature, however previous studies had mainly focused on prokaryotes to investigate polyP metabolism. PolyP is a polyanion consisting of up to several hundreds of phosphate units (P_i_) linked by energy-rich phosphoanhydride bonds. PolyP is formed in bacteria by polyP kinase through transfer of ATP/GTP's γ-phosphate residues onto the nascent polymer chain (1, 2), while depolymerization of polyP is catalyzed by exopolyphosphatase (ppx) (3). Dependency of polyP for bacterial growth and survival initiated efforts to develop drugs that target polyP metabolism [reviewed in (4–6)].

Eukaryotic polyP metabolism is poorly understood with exception for *S. cerevisiae* cells. Yeast expresses a polyP polymerase, vacuolar transporter chaperone, and polyP phosphatases including exopolyphosphatase (Ppx1), endopolyphosphatases (Ppn1) and diadenosine and diphosphoinositol phosphohydrolase (Ddp1) (7–10). Despite intensive investigations, mammalian homologs for these polyP-related enzymes have not been identified, however diphosphoinositol polyP phosphohydrolases (DIPPs) have been shown to degrade polyP in alkaline conditions (10). The same report also demonstrated that intracellular polyP concentrations were dependent on enzymes regulating inositol phosphorylation, such as phospholipase C, inositol polyP multikinase and inositol hexakisphosphate kinase (Ip6k1) in yeast (10). Notably, genetic ablation of *Ip6k1*, a gene coding for the kinase that generates diphospho-moieties through phosphorylation of the 5-position of inositol penta- and hexakisphosphate, has been shown to reduce platelet polyP levels in mice (11). Together, the data suggest that polyP is intertwined with polyphosphorylated inositol metabolism. Multiple cellular processes are regulated by the (poly)phosphorylated inositol, including signal transduction, Ca^2+^ channel permeability and gene expression [reviewed in (12)]. Particularly, immune cell activation depends on membrane-bound phosphoinositides and soluble inositol phosphates [reviewed in (13)].

In yeast, intracellular concentrations of P_i_ are sensed through P_i_-responsive signaling (Pho-regulon) mediated by both, polyP and diphosphoinositol pentakisphosphate (14, 15). Under phosphate-limiting growth conditions, the transcription factor Pho4 controls expression of high affinity P_i_ transporters and secreted acid phosphatases to replenish intracellular P_i_. Orthologs of the yeast Pho-regulon seem not to exist in multicellular eukaryotes. However, P_i_ sensor domains have been identified as part of P_i_ transporters that are conserved among various organisms and share a SPX domain (16). This common SPX domain, named after yeast Syg1 and Pho81 and human XPR1 (xenotropic and polytropic retrovirus receptor 1), binds to diphosphoinositol pentakisphosphate and increases polyP synthesis in yeast and plants. Mice with conditional deficiency of Xpr1 in renal tubular cells develop proximal tubular dysfunction (17). In humans, impaired XPR1 function due to mutations in the P_i_ exporter is associated with primary familial brain calcification (18). These findings indicate a link between P_i_ sensing, intracellular P_i_ levels and polyP, although further studies in eukaryotes are required to elucidate polyP regulation *in vivo*.

## Intracellular Roles of Polyphosphate

High cytoplasmic polyP levels have been found in various cell lines, including NIH3T3 fibroblasts, Vero epithelial kidney cells and Jurkat CD4+ T cells (19). Tissues with high energy demand and high regeneration or proliferation capacity (e.g., brain, heart, liver, and cancer cells) are rich in polyP (19–21). In line with polyP's function as energy storage pool, defective polyP synthesis confers disadvantages for growth and survival of bacteria, fungi and protozoa (9, 22, 23). In bacteria, polyP is bound to Ca^2+^ ions and complexed with poly-β-hydroxybutyrate forming membrane channels utilized for DNA uptake (24, 25).

In eukaryotes, polyP has functions in mitochondria and the polymer is enriched within the intermembrane compartment and uncouplers of oxidative phosphorylation decrease mitochondrial polyP levels in *S. cerevisiae* (26, 27). Degradation of polyP using Ppx1 polyphosphatase, that is localized to the mitochondrial intermembrane space by a cytochrome c-derived targeting sequence, decreases the membrane potential and increases NADH levels in mitochondria (28, 29). Moreover, polyP appears to contribute to opening of the mitochondrial permeability transition pore in cardiac myocytes. In these cells in culture, Ppx1-mediated polyP degradation inhibits mitochondrial Ca^2+^ ion accumulation and interferes with Ca^2+^-induced cell death that is associated with myocardial infarction and ischemia reperfusion injury (30).

Based on the function of the polymer for growth and survival, polyP is believed to have a role in malignant diseases. In cancer cells, polyP has been detected in epithelial tumor cell lines derived from prostate and mammary gland as well as in primary myeloma B cells (21, 31, 32). In addition to cancer-associated pro-coagulant activities of extracellular polyP (see below), the polymer facilitates various intracellular functions in cancer cells. PolyP increases the kinase activity of mammalian target of rapamycin (mTOR) in MCF-7 tumor cells and accumulates at nucleolar transcription sites in myeloma cells. In contrast, Ppx1-mediated polyP degradation and actinomycin D-induced transcription inhibition abrogate these polyP effects (31, 32). Together, the data suggest, that polyP may act as metabolic driving force in different cellular compartments, thereby promoting tumorigenesis.

As a negatively charged polymer, polyP exerts chaperone activity in bacteria such as *E. coli* (33, 34). Short and long chain polyP regulate ribosomal translation efficiency and ribosomal protein degradation, indicating a differential role of polyP dependent on chain lengths in bacterial protein biosynthesis (35, 36). Similarly to the polymer starch and its monomeric form glucose, polyP is condensed phosphate and reduces the concentration of P_i_ and thus the intracellular osmotic pressure conferred by the ion. The polymer acts as P_i_ storage pool and serves as non-enzymatic protein (pyro-)phosphorylation mediator (37, 38) [as does inositol pyrophosphate (39, 40)] in eukaryotes. PolyP also activates the cytoplasmic portion of transient receptor potential (TRP) A1 and melastatin 8 in neurons (41, 42). Moreover, a recent report identified a glucokinase that relies on polyP as phosphoryl donor in the liver (43), indicating that future research on tissue-specific polyP functions may broaden our view of its physiological roles in various cell types. In this context, Ca^2+^-dependent, mTOR- and TRP-regulated immune responses could be a potential target for polyP.

Besides its established mitochondrial and nuclear distribution, polyP accumulates in intracellular vesicles. Yeast, parasites, mast cells and platelets store polyP in vacuoles, acidocalcisomes, heparin-containing granules and dense granules, respectively (44–48). Ca^2+^-complexed polyP derived from these acidic organelles is insoluble, retained on the plasma membrane in nanoparticle form upon release and provides the pro-coagulant surface for factor XII (FXII) contact activation (49). PolyP-containing vesicles are also found in lysosome-related organelles derived from fibroblasts and astrocytes (50, 51). The latter cell type releases polyP as a neurotransmitter from lysosomes expressing vesicular nucleotide transporter in response to pH changes, exogenous polyP and Ca^2+^ ion signaling (52). Taken together, these studies indicate that polyP have fundamental functions for secretion and possibly stability of lysosome-related vesicles in other cell types, such as immune cells. However, quantification of P_i_ via malachite green assay and ^32^P_i_ via radiodetection obtained from hydrolysed polyP has to be interpreted with caution, since microbial contaminations interfered with these assays to detect polyP in human granulocytes in a previous report [update to (53)]. Analysis of *in vivo* polyP functions is an area of ongoing research and recently a flow cytometry-based assay has been established to quantify the polymer on cell surfaces (54). Table 1 provides a summary of polyP functions in different organisms, cell populations and intracellular compartments.

**Table 1:** Overview on polyP-mediated activities in various cell types.

**Organism**	**Location**	**PolyP-function**	**Effect**	**References**
Bacteria	Various phyla	PolyP metabolism	Increased virulence	(22)
		Membrane pore formation	Vector uptake	(25)
		Protein biosynthesis	Expression control, chaperone activity	(33–36)
Fungi	*S. cerevisiae*	P_i_ sensing	P_i_ regulon	(16)
		P_i_ metabolism	P_i_ reservoir	(37)
		Mitochondrial energy storage	Supported oxidative phosphorylation	(27)
	*D. discoideum*	PolyP secretion	Growth inhibition	(55, 56)
Animals	Amoeba histolyticum	PolyP metabolism	Increased biological fitness	(23)
	Osteoblasts	Mineralization inhibition	Apatite binding	(57)
	Myocytes	Mitochondrial permeability transition pore activation	Ca^2+^ ion accumulation	(30)
	Hepatocytes	Metabolic contribution	Metabolic control	(28, 43)
	Neurons	TRPA1, TRPM8 signaling	Stimulating co-factor	(41, 42)
	Astrocytes	Vesicular release	Neurotransmitter	(51, 52)
	Fibroblasts	Fibroblast growth factor binding	Unknown	(50, 58, 59)
	Epithelial cells	mTOR pathway	Proliferation	(31)
	Endothelial cells	mTOR, P2Y_1_, and Wnt pathways	Induced apoptosis, permeability, cell adhesion	(60–63)
	Platelets	PolyP secretion	Bradykinin formation	(48)
	Platelets	PolyP secretion	FXIIa-mediated coagulation	(48, 64)
	Platelets	Extracellular nuclear protein binding	Increased vascular permeability	(62)
	Mast cells, basophils	PolyP secretion	Bradykinin formation	(46)
	Neutrophils	mTOR inhibition, autophagy induction	NET formation	(65)
	Plasma B cells	Unknown mechanism	Apoptosis	(66)
	Plasma	Increased C1 esterase inhibitor activity	Matrix for C1 esterase inhibitor regulation	(67)
	Plasma	Complement system	Inhibition	(68)
	Plasma	Platelet factor 4 binding	Autoimmune-induced thrombocytopenia	(69)

## Extracellular Polyphosphates

While intracellular polyP activities are established only recent data have revealed a role of extracellular polyP in mammalian and human cardiovascular biology. The *in vivo* activation of FXII in the initiating steps of the intrinsic blood coagulation system has been an enigma for many years [reviewed in (70)]. FXII activation by binding to negatively charged kaolin or ellagic acid (“contact activation”) provides the mechanistic basis for activated partial thromboplastin time (aPTT), the clinical coagulation test (with an estimated 4–5 billion tests annually). However, the natural surface that induces FXII contact activation *in vivo* had been unknown. Activated platelets induce plasma clotting in a FXII-dependent manner, indicating that platelets release FXII-activating structures. Studies in mice and human plasma revealed that polyP serves as the long sought FXII-activating surface on activated platelets linking primary and secondary hemostasis. *Vice versa*, humans with polyP deficiency (Hermansky Pudlak Syndrome) have defective platelet-driven FXII activation and clotting [(48) and reviewed in (71) and (72)]. In addition to FXII activation, polyP has been reported to modulate other coagulation reactions *in vitro*, however a potential role of these pathways *in vivo* remains to be demonstrated (64, 73–75). The chain length of synthetic polyP determines its solubility and FXII activation capacity in plasma (76). However, natural platelet polyP forms insoluble Ca^2+^ ion-rich nanoparticles independently of the chain length of the polyP molecules that are maintained on the surfaces of pro-coagulant platelets and thus activate FXII (49). Hence size of polyP does not matter for FXII-activating potency of the physiologically occurring Ca^2+^-saturated polymer. Similarly to polyP, also synthetic polyI:C, a dsRNA analog that activates Toll-like receptor 3, has been shown to have pro-coagulant activity (77).

PolyP is a chelator for positive metal ions and dense granules of platelets contain polyP bound to Ca^2+^ and possible Zn^2+^ ions at high concentrations (47). In plasma, Ca^2+^-saturated polyP has a half-life of about 90 min (76), due to degradation by polyphosphatases, such as alkaline phosphatase that has exopolyphosphatase activity (48, 78). Synthetic polyP derived from melted phosphoric acid is mostly complexed to Na^+^ ions. Counterions regulate structure, biophysical properties and biological activities of polyP (49). Most of the *in vitro* studies have been performed with synthetic (often Ca^2+^-free) polyP in the absence of phosphatases. Physiologically occurring Ca^2+^-polyP is not soluble and operates on the cell surface. The low solubility in plasma challenges suggested functions for the extracellular polymer in solution. In contrast Ca^2+^-free synthetic polyP of short and medium sized chain length (50–150 P_i_ moieties) depletes free Ca^2+^ ions leading to anti-coagulant effects similarly to EDTA (76).

Most cell culture experiments have been performed with medium sized synthetic polyP (10–50 μM, based on monophosphate units). In buffer or plasma environments *in vitro*, synthetic polyP is associated with an array of binding partners, including von Willebrand factor, coagulation factors XII, V and XI, complement factors C1s, C5b6, C6, and C7 (64, 68, 73, 74). Medium chain polyP and heparin have been reported to bind C1 esterase inhibitor (C1INH), thereby increasing function of the inhibitor (67, 79). However, FXII is readily activated by platelet-derived polyP to initiate the intrinsic coagulation cascade *in vivo*, indicating that increased activity of physiologic C1INH levels following polyanion binding is not sufficient to prevent FXIIa-driven coagulation. In contrast, high dosages of C1INH (15 IU/kg) but not low dosages (7.5 IU/kg) decrease ischemia/reperfusion injuries (80), suggesting that polyP binding to supra-physiological C1INH levels has the capacity for regulating thrombosis. The amount of polyP exposed on activated platelet surface is not precisely known, and it remains to be analyzed whether low amounts of platelet-derived C1INH that is retained on the surface of activated platelets and is possibly bound to polyP have a role in regulating serine proteases *in vivo* (81).

PolyP increases endothelial cell permeability *in vivo*, stimulates expression of cell adhesion molecules and induces apoptosis in culture (60). Follow-up studies, have elucidated the pathways involved in these endothelial reactions. PolyP application activates NF-κb and β-catenin pathways via receptor for advanced glycation end products, P2Y_1_ receptor signaling and Wnt signaling (61, 62). *Vice versa*, the anti-thyroid drug methylthiouracil interferes with polyP-mediated inflammation of the endothelium *in vivo* (82). On the other hand, probiotic-derived polyP improves epithelial barrier function and may contribute to immunomodulatory mechanisms against commensal bacteria in the gut via NF-κb inhibition (83).

High extracellular polyP levels (>500 μM) elicit scaffolding effects that control amyloidogenic processes (84). In addition, polyP binds to fibroblast growth factor (FGF) (58). However, possible effects of polyP-FGF complexes on FGF-receptor signaling have remained controversial, as both increased and inhibited mitogenic activities have been reported (58, 59). A possible explanation for the dual activities might be that FXII, which initially promotes angiogenesis via urokinase plasminogen activator receptor (uPAR) signaling (85), gets rapidly depleted following polyP application via binding of FXIIa to C1INH. In the presence of serum-derived C1INH, FXIIa gets rapidly cleared and might limit FXII(a)/uPAR-driven pathways. Supporting this idea, tumor cells secrete exosomes that expose polyP and are associated with worse outcome involving immune evasion possibly through reduced FXII(a)/uPAR-driven differentiation of tumor-reactive T cell subsets (21, 86, 87). Moreover, human plasma cells induce apoptosis following polyP treatment, whereas other lymphocyte populations do not (66).

Presentation of polyP on activated platelets allows for contact of polyP with immune cells. Activation of platelets increases the intracellular Ca^2+^ ion concentration via stromal interaction molecule 1 to release Ca^2+^ ions from the sarcoplasmatic reticulum (88), which opens store-operated calcium entry channels in the plasma membrane (89). Ca^2+^-mediated platelet activation mirrors the process of T cell stimulation (90). Moreover, conjugates of platelet-CD4+ T cells have been reported (91) and T-cell proliferation and differentiation is modulated by platelet-released molecules, such as platelet factor 4 (92), soluble CD40 ligand (93), ADP/ATP (94) and transforming growth factor-β (95). Recent findings also suggest that platelets inhibit pro-inflammatory interleukin (IL)-17 secreting effector T helper (Th17) cells in a tumor model (96). Taken together, these studies suggest that polyP modulates immune responses via direct or indirect pathways.

Neutrophils express FXII that is translocated to the plasma membrane upon activation and facilitates uPAR signaling via an autocrine mechanism (97). FXII signaling in neutrophils is independent of FXII cleavage and promotes adhesion, migration and formation of neutrophil extracellular traps (NET). In transfer experiments, neutrophils derived from FXII-deficient bone marrow show reduced tissue infiltration and better wound healing in the host, compared to neutrophils derived from wild-type bone marrow. PolyP treatment of neutrophils *in vitro* is independent on uPAR signaling and promotes the generation of NET through decreased mTOR acitivity and autophagy induction (65). Supporting a link between polyP and neutrophils, neutrophil-mediated inflammation is reduced in polyP-deficient mice in two independent mouse models with (i) genetic *IP6K1* ablation or (ii) pharmacological IP6K1 inhibition (98). The data suggest that both neutrophil FXII and platelet-derived polyP contribute to NET formation.

Both polyP and heparin are negatively charged polymers and polyP has been shown to substitute for heparin. Both heparin and polyP derived from mast cells drive the activation of the kallikrein-kinin system (46, 99). Antibodies that recognize complexes of platelet factor 4 (PF4) bound to heparin induce platelet aggregation in heparin-induced thrombocytopenia (HIT). These antibodies also cross-react with PF4/polyP complexes (69), indicating that polyP might contribute to HIT offering a rational for the recently described heparin-independent HIT forms (100).

## Polyphosphate-Mediated Inflammation

PolyP promotes the autocatalytic activation of FXII to the serine protease FXIIa and FXIIa production is amplified by the protease plasma kallikrein (PKa) through a feed-forward mechanism of FXIIa-cleaved plasma prekallikrein (PK, fluid phase activation). Deficiency in FXII is not associated with obvious abnormalities in humans and mice. However, FXII-deficient plasma (Hageman trait) has a prolonged aPTT. Mice lacking FXII (F12^−/−^) have largely defective arterial and venous thrombus formation (101) and are protected from thromboembolic diseases without showing hemostatic abnormalities (48, 102). The impact of FXII on thrombosis recently steered the World Health Organization to establish an international standard for human plasma-derived FXII (103). The fact that FXII deficiency is not associated with bleeding, suggests that FXII-mediated intrinsic blood coagulation contributes to other vascular pathways rather than hemostasis [reviewed in (104)].

FXII and FXIIa are both ligands of uPAR that is expressed on antigen-presenting cells and promotes differentiation of pro-inflammatory Th17 cells (85, 87). Consistently, F12^−/−^ mice were shown to be protected from Th17-driven experimental autoimmune encephalomyelitis (EAE) development (87). Autoimmune diseases, such as EAE, require the extravasation of auto-reactive Th1 and Th17 cells to infiltrate and attack target cell structures. Furthermore, FXIIa increases vascular permeability via the kallikrein-kinin system, in which bradykinin (BK) is produced by PKa-mediated high molecular weight kininogen cleavage. BK is a peptide hormone and BK signal transduction is mediated by G-coupled kinin B1 and B2 receptors on endothelial cells as well as leukocytes [reviewed in (105)]. Kinin B1 receptor expression is induced by pro-inflammatory cytokines (e.g., IL-1β) and stimulated by BK and the C-terminal truncated BK derivate des-Arg(9)BK, whereas constitutively expressed kinin B2 receptors recognize BK [reviewed in (106)]. Deficiency in kinin B1 receptors affects Th-cell migration and outcome in murine EAE models and possibly humans with multiple sclerosis (107), suggesting that several FXII-dependent pathways can interact with T-cell homeostasis. In line with this notion, increased populations of Th17 cells have been reported in patients with defective FXIIa regulation (108).

**Figure 1:**
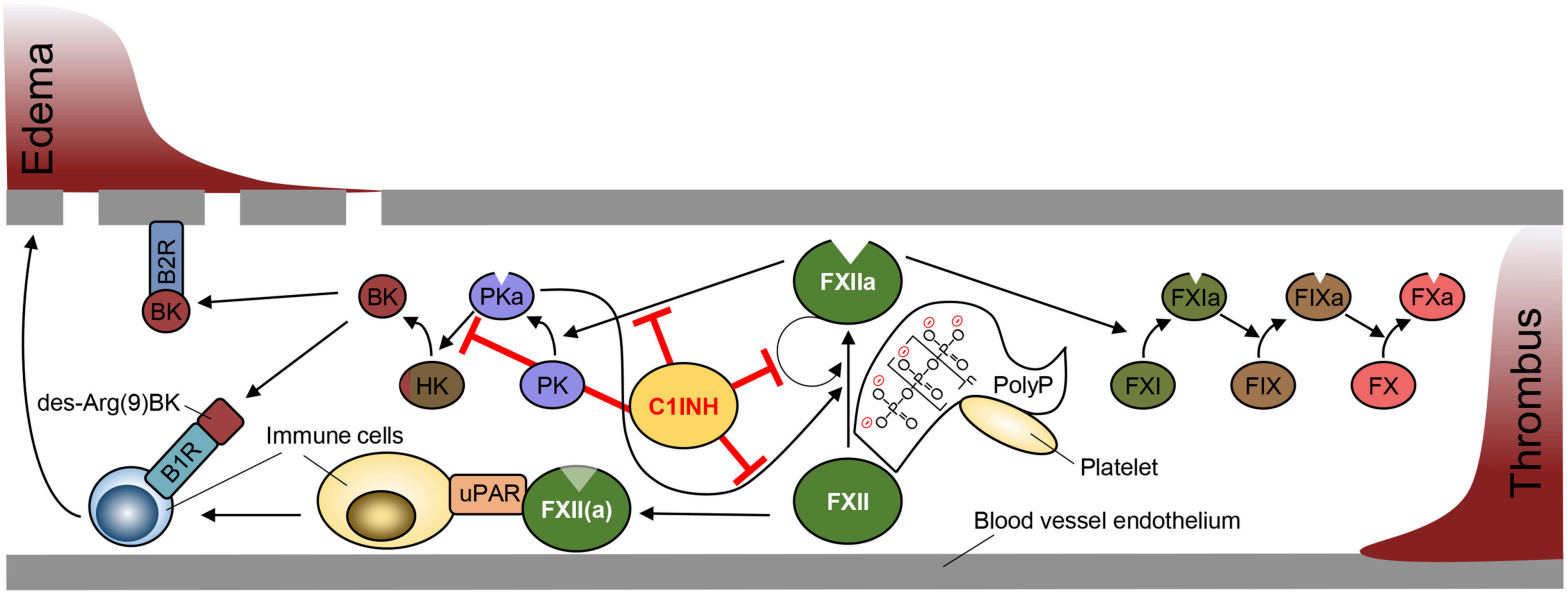
Schematic overview of Factor XII-mediated pathways in edema and thrombosis formation. Factor XII (FXII) activation is driven by autocatalytic activation promoted by binding to negatively charged surfaces (contact activation), such as polyphosphate (PolyP) retained on platelet surface. FXIIa production is amplified by plasma kallikrein (PKa) that is produced from FXIIa-cleaved plasma prekallikrein (PK). C1 esterase inhibitor (C1INH) blocks the activity of FXIIa and PKa to prevent PKa-driven edema formation via cleavage of high molecular weight kininogen (HK) to release bradykinin (BK). Binding of BK and its metabolite des-Arg(9)BK to bradykinin receptors (B1R, B2R) promotes vascular permeability and immune cell activation. FXII and FXIIa bind urokinase plasminogen activator receptor (uPAR) to stimulate immune responses (left). FXIIa initiates the activation of the intrinsic coagulation cascade via activation of factor XI (FXI), factor IX (FIX) and factor X (FX) to form a thrombus (right).

Proteolytic activity of FXIIa is regulated by C1INH, a serpin that irreversibly inhibits FXIIa through covalent binding into its reactive center. C1INH also has the capacity to inhibit several other serine proteases, including PKa, active factor XI (FXIa) and complement factors C1r and C1s. Impaired FXIIa inhibition augments BK formation by the kallikrein-kinin system and is associated with a BK-mediated life-threatening inherited swelling disorder, hereditary angioedema (HAE) [reviewed in (109)]. C1INH deficiency has no impact on thrombosis and HAE patients have a normal thromboembolic risk [reviewed in (110)]. HAE is a rare disease that is mainly autosomal dominant inherited and characterized by reduced C1INH levels (HAE type I) or function (HAE type II). In addition HAE has been reported in patients with normal C1 esterase inhibitor activity (HAE type III). Disease-causing HAE type III mutations have been mapped to the *F12* gene. Analysis of mutant FXII T309K and T309R (positions refer to the mature protein) revealed that mutant FXII is defective in a single O-linked glycosylation, which promotes contact-driven FXIIa formation (111). Additionally, the mutations create new serine protease cleavage sites and thus increase FXIIa- and plasmin-mediated mutant FXII zymogen activation (112). Other HAE type III associated mutations have been linked to plasminogen and angiopoeitin (113, 114). While BK is short-lived and degraded within seconds, the activity of FXIIa or activation of FXII is increased in HAE leading to sustained and prolonged BK formation (115, 116). A schematic overview depicting PolyP/FXII-mediated pathways is shown in Figure 1.

In addition to the kallikrein-kinin system, FXIIa triggers the intrinsic blood coagulation pathway that has a role in thrombus formation [reviewed in (117)]. Thrombi occlude vessels causing tissue ischemia but also function in host defense in a concept termed “immunothrombosis.” A recent report on bacterial sepsis-associated pro-coagulant mechanisms showed that inflammation-driven thrombosis occurs earlier in the spleen compared to liver despite similar bacterial burden in both organs, suggesting that organ-specific environment rather than bacterial components contribute to microthrombus formation (118). Bacteria-derived polyP is highly pro-coagulant *in vitro*, however, it is unknown whether bacteria have the capacity to expose or secrete their long chain polyP (>1,000 P_i_ moieties).

**Figure 2:**
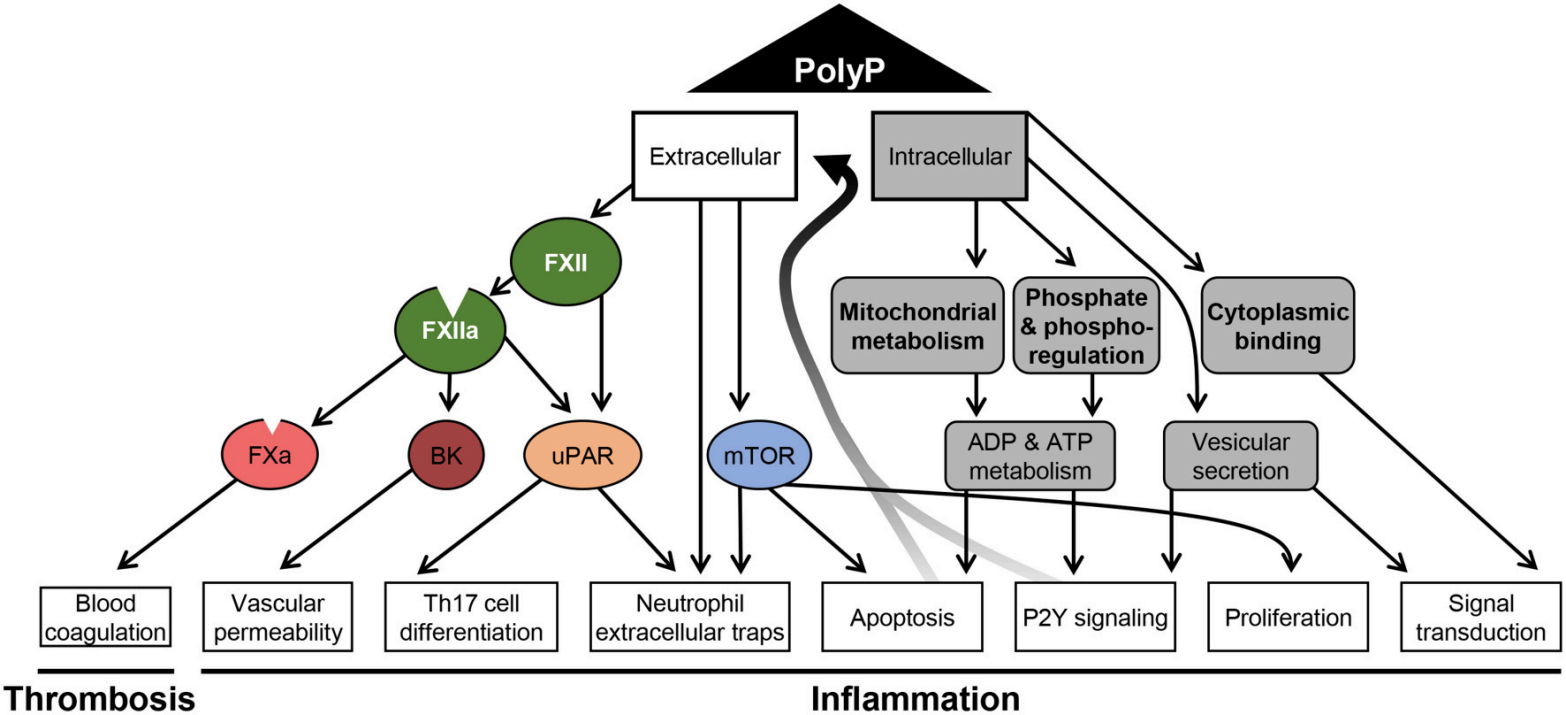
Schematic overview of polyphosphate-mediated pathways. A summary of the diverse mechanisms that are affected by polyP and have been reported for distinct cell types is shown. Polyphosphate (polyP) regulates intracellular mechanisms (gray) related to metabolism, signaling and apoptosis. The effect of polyP on phoshoregulation has been shown in yeast, the impact on mitochondrial activity in yeast and cardiomyocytes. Protein binding and vesicular secretion of polyP was reported for neurons and polyP-driven proliferation has been shown for tumor cells. Active and passive release of polyP promotes extracellular pathways to activate coagulation factors (FXIIa and FXa), to generate bradykinin (BK) and to stimulate cells via urokinase plasminogen activator receptor (uPAR)- and mammalian target of rapamycin (mTOR)-driven mechanisms. Signaling via these mediators were reported to affect endothelial cells and immune cells.

Intracellular polyP gets released during necrosis and may act as a damage associated molecular pattern inducing immune responses through P2Y signaling (62). Indeed, long chain polyP induces thrombosis via FXII activation but also leads to platelet activation and consumptive coagulopathy (119). Moreover, polyP serves as phosphoryl donor for adenylate kinase in the extracellular space (120). Thus, polyP may regulate ADP/ATP ratio and signaling via purinergic receptors in immune cells. An overview of the complex network by which polyP impacts on cellular mechanisms in different cell types to facilitate coagulation and inflammation is depicted in Figure 2.

## Interference with Polyphosphate Functions

Intracellular polyP levels have been modulated in *S. cerevisiae* through phosphate starvation and abrogation of the mitochondrial membrane potential by uncouplers (27), indicating that intracellular polyP broadly influences cell metabolism. Moreover, Ppx1-mediated degradation of polyP regulates mitochondrial Ca^2+^ ion efflux (30).

Mammalian enzymes that control formation and degradation of polyP have remained unknown. To interfere with polyP/FXIIa-driven inflammation plasma derived C1INH (Berinert™) and B2R antagonist (Icatibant™) dampen bradykinin formation and signaling and have been used for treatment of excessive bradykinin-mediated swellings in HAE patients (121, 122). Neutralizing FXIIa antibody 3F7 prevents thromboembolism and bradykinin generation with minimal therapy-associated adverse effects such as increased bleeding (123). Interference with the extracellular polyP/FXII pathway is a promising strategy to dampen coagulation and inflammation [reviewed in (124)]. To directly target extracellular polyP, neutralization and degradation approaches have been tested. Structural homology of polyP and heparin suggests that the polycation protamine sulfat, the heparin antidote, can also neutralize polyP. Indeed, polyP inhibitor screening identified various polycation substrates, such as spermidine, histone H1, polymyxin B and cationic polymers as possible agents for interference with polyP-mediated coagulation and inflammation (125). Moreover, the recombinant polyP-binding domains of *E. coli* ppx (PPX_Δ12) bind to polyP with high affinity and block polyP-mediated FXII activation. Similarly, degradation of polyP with recombinant ppx inhibits arterial and venous thrombus formation without interfering with hemostasis, indicating that polyP operates via FXII in *in vivo* coagulation (126).

Interference with polyP developed evolutionary in blood-sucking insects. Sand flies express salivary proteins that enable the insects to feed on mammalian blood without triggering BK-mediated itching or FXIIa-mediated clotting. These proteins, termed PdSP15a and PdSP15b, provide a positively charged helix that efficiently binds to and neutralizes polyP (127). Taken together, polyP inhibition has been shown to block thrombosis and inflammation and provides an opportunity for efficient and safe future treatment.

## Outlook

Recent advantages to visualize, measure and detect polyP and to modulate polyP metabolism offers the possibility to analyze cell-specific roles for polyP (11, 28, 126, 128). Open research questions include regulation of polyP in mammalians and eukaryotic cells, and possibilities to target polyP-mediated activities in therapeutic settings.

## Author Contributions

RM wrote the manuscript and created the figures. LH, MA, and TR reviewed the manuscript.

### Conflict of Interest Statement

The authors declare that the research was conducted in the absence of any commercial or financial relationships that could be construed as a potential conflict of interest.
